# Arm-specific dynamics of chromosome evolution in malaria mosquitoes

**DOI:** 10.1186/1471-2148-11-91

**Published:** 2011-04-07

**Authors:** Maria V Sharakhova, Ai Xia, Scotland C Leman, Igor V Sharakhov

**Affiliations:** 1 Department of Entomology, Virginia Tech, Blacksburg, VA 24061, USA; 2 Department of Statistics, Virginia Tech, Blacksburg, VA 24061, USA; 3 Current Address: Entomology department, Nanjing Agricultural University, Nanjing, 210095, P.R. of China

## Abstract

**Background:**

The malaria mosquito species of subgenus *Cellia *have rich inversion polymorphisms that correlate with environmental variables. Polymorphic inversions tend to cluster on the chromosomal arms 2R and 2L but not on X, 3R and 3L in *Anopheles gambiae *and homologous arms in other species. However, it is unknown whether polymorphic inversions on homologous chromosomal arms of distantly related species from subgenus *Cellia *nonrandomly share similar sets of genes. It is also unclear if the evolutionary breakage of inversion-poor chromosomal arms is under constraints.

**Results:**

To gain a better understanding of the arm-specific differences in the rates of genome rearrangements, we compared gene orders and established syntenic relationships among *Anopheles gambiae, Anopheles funestus*, and *Anopheles stephensi*. We provided evidence that polymorphic inversions on the 2R arms in these three species nonrandomly captured similar sets of genes. This nonrandom distribution of genes was not only a result of preservation of ancestral gene order but also an outcome of extensive reshuffling of gene orders that created new combinations of homologous genes within independently originated polymorphic inversions. The statistical analysis of distribution of conserved gene orders demonstrated that the autosomal arms differ in their tolerance to generating evolutionary breakpoints. The fastest evolving 2R autosomal arm was enriched with gene blocks conserved between only a pair of species. In contrast, all identified syntenic blocks were preserved on the slowly evolving 3R arm of *An. gambiae *and on the homologous arms of *An. funestus *and *An. stephensi*.

**Conclusions:**

Our results suggest that natural selection favors specific gene combinations within polymorphic inversions when distant species are exposed to similar environmental pressures. This knowledge could be useful for the discovery of genes responsible for an association of inversion polymorphisms with phenotypic variations in multiple species. Our data support the chromosomal arm specificity in rates of gene order disruption during mosquito evolution. We conclude that the distribution of breakpoint regions is evolutionary conserved on slowly evolving arms and tends to be lineage-specific on rapidly evolving arms.

## Background

Despite the growing recognition of the importance of chromosomal inversions for adaptation and evolution of species [1-4], the evolutionary forces responsible for rearrangement establishment and maintenance remain an enigma of evolutionary biology. Comparative mapping has yielded important insights into patterns and mechanisms of genome rearrangements in plants, mammals, fruit flies, and yeasts [5-10]. One of the important findings is that inversions are distributed nonuniformly across different chromosomes. Cytogenetic studies performed on malaria mosquito species of subgenus *Cellia *provided some of the most obvious examples of the nonuniform inversion distribution [11-14]. *Cellia *is the largest subgenus within genus *Anopheles *and is restricted to the Old World. It includes some of the most important malaria vectors, such as, *An. gambiae, An. funestus*, and *An. stephensi*. The polytene chromosome complement of *Anopheles *female consists of the X chromosome and four autosomal arms: 2R, 2L, 3R, and 3L. A study of the distribution of 82 polymorphic inversions in *An. gambiae s.s. *has found that the chromosome 2 is the most inversion-rich. It has 77 inversions versus only five inversions on chromosome 3 [13]. In *An. funestus *and *An. stephensi*, polymorphic inversions tend to cluster on the chromosomal arms that are homologous to the 2R and 2L arms of *An. gambiae *[15-18]. These observations suggest that genome rearrangements have chromosome-specific facilitators or inhibitors. The major consequence of unequal rates of chromosome evolution is a disproportionally large role of genetic content of certain chromosomal arms in adaptation and, possibly, speciation. However, the mechanisms that govern the unequal distribution of rearrangements among chromosomes are poorly understood.

The common inversions *2Rb, 2Rbc, 2Rcu, 2Ru, 2Rd*, and *2La *of *An. gambiae s.s. *are widespread in the arid sub-Saharan Africa but almost absent in the humid equatorial Africa [14]. Similarly, multiple inversions on arms 2R and 3R in natural populations of *An. funestus *are fixed in the southern humid rainforest area of Cameroon and decrease in frequency going northwards, with their complete absence in the northernmost dry Sahelian savannas [19]. Chromosomal studies of the Asian malaria vector *An. stephensi *reveal striking differences in the kinds and frequencies of paracentric inversions on 2R and 3L between rural and urban populations, especially with respect to the common *2Rb *inversion [16]. These observations suggest that the genes on the 2R and 2L arms of *An. gambiae *and on the homologous arms of *An. funestus *and *An. stephensi *are more sensitive to variation in the environment experienced by these species and thus show evidence of selective response to environmental pressures. More recent studies provided ecological evidence that sympatric species *An. gambiae *and *An. funestus *inhabit a wide range of the same ecoclimatic settings in Cameroon [20,21], suggesting an intriguing possibility that polymorphic inversions capture identical sets of genes in different species and, thus, confer similar ecological adaptations. It has been proposed that inversions *2st *of *D. buzzatii *and *In(3R)Payne *of *D. melanogaster *have common genes because both inversions are associated with body size [22]. However, the nonrandom occurrence of similar sets of genes within polymorphic inversions of different species has not yet been demonstrated. The presence of common genes within inversions of homologous chromosomal arms would indicate that natural selection favors certain adaptive gene combinations when different species are exposed to similar environmental variables.

Polytene chromosomes, which are present in many species of *Anopheles*, provide an opportunity to develop high-resolution physical maps and to study genome organization and evolution in malaria mosquitoes. We previously reported a 1-Mb resolution physical map for *An. stephensi *and analyzed the evolutionary dynamics of chromosomal inversions in subgenus *Cellia *[11]. The study has shown that despite the paucity of inversion polymorphisms on the X chromosome, this chromosome has the fastest rate of inversion fixation, while the 2R arm has the highest inversion fixation rate among autosomes. The results have also indicated that the rapidly and slowly evolving chromosomal arms have different genome landscapes characterized by distinctly enriched gene subpopulations and classes of repetitive DNA. Although the propensity of chromosomes to rearrangements seems to play a major role in the rates of inversion origin, negative and positive selections may differentially control the establishment and maintenance of polymorphic inversions on different chromosomal arms. For example, chromosome arms 3R and 3L of *An. gambiae *and homologous arms in other mosquito species have the lowest rates of inversion polymorphism and fixation [11,13,16,18]. The low tolerance of a chromosome to gene order disruption could contribute to the low rate of inversion establishment. Large blocks of genes that are conserved in the evolution of several species may represent functionally important combinations that are maintained together by natural selection [23] and/or genomic fragments devoid of evolutionary fragile regions [24]. Comparative analysis of gene order preservation and disruption across species can determine possible differences in the chromosomal arm tolerance to rearrangements.

Here, we report evolutionary insights from a comparative physical mapping among three species of malaria mosquitoes. We demonstrated that polymorphic inversions on the 2R arm, involved in environmental adaptations in these three species, nonrandomly share similar sets of genes. These results suggest that distantly related species acquire parallel adaptations through capturing common genes by independent polymorphic inversions. We also found that the chromosomal arms differ in their tolerance to gene order disruption during mosquito evolution. The distribution of breakpoint regions is evolutionary conserved on slowly evolving arms and tends to be lineage-specific on rapidly evolving arms. The arm-specific tolerance to chromosomal breakage could be responsible for the nonuniform establishment of inversions.

## Results

### Inversion distances among *An. stephensi, An. gambiae*, and *An. funestus*

**Figure 1:**
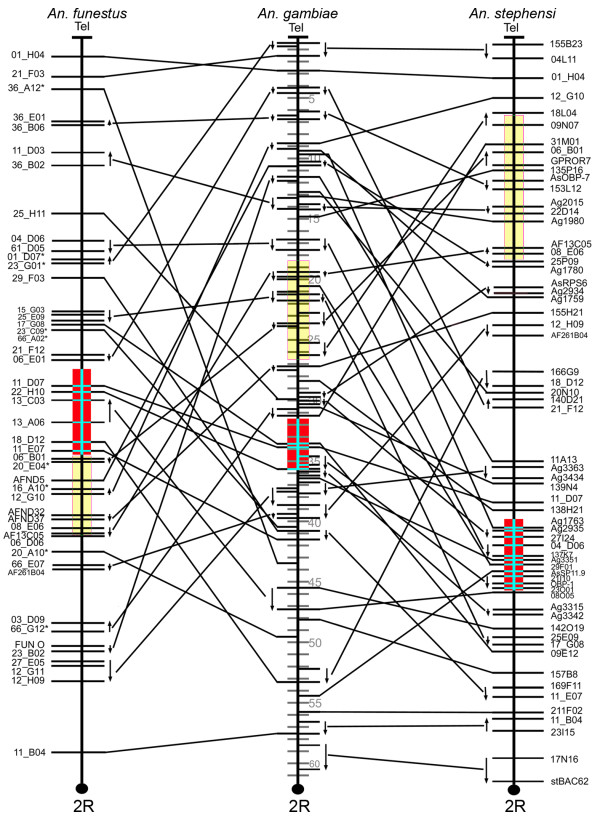
**Comparative mapping of chromosomal arms 2R of *An. gambiae, An. funestus*, and *An. stephensi***. Arrows denote oriented conserved gene orders. The red boxes indicate positions of polymorphic inversions *2Rt *of *An. funestus, 2Ru *of *An. gambiae*, and *2Rf *of *An. stephensi*. The yellow boxes indicate positions of polymorphic inversions *2Rd/2Rh *of *An. funestus, 2Rb *of *An. gambiae*, and *2Re *of *An. stephensi*. Shaded divisions on the *An. gambiae *chromosomes denote the genomic coordinates in this species. The centromere regions are shown by black circles at the end of the arms.

**Figure 2:**
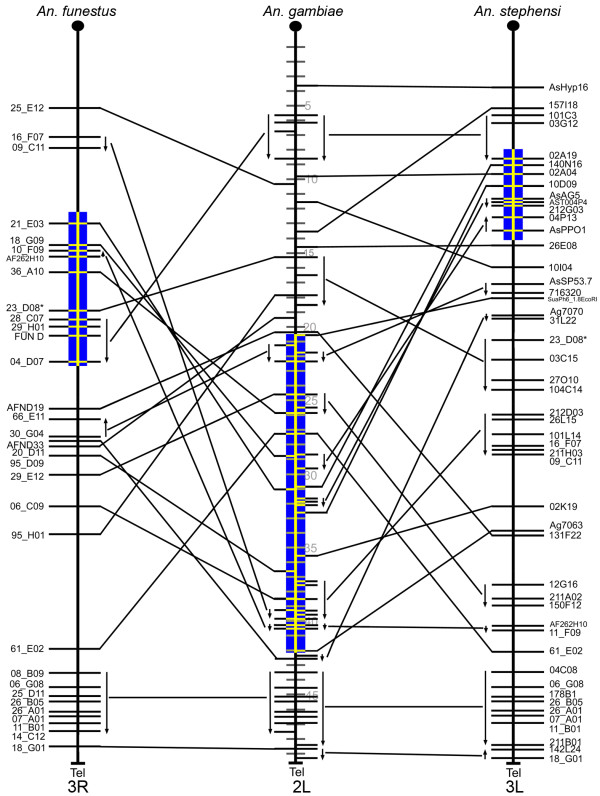
**Comparative mapping of chromosomal arms 2L of *An. gambiae*, 3R of *An. funestus*, and 3L of *An. stephensi***. Arrows denote oriented conserved gene orders. The blue boxes indicate positions of polymorphic inversions *3Rb *of *An. funestus, 2La *of *An. gambiae*, and *3Lf *of *An. stephensi*. Shaded divisions on the *An. gambiae *chromosomes denote the genomic coordinates in this species. The centromere regions are shown by black circles at the end of the arms.

**Figure 3:**
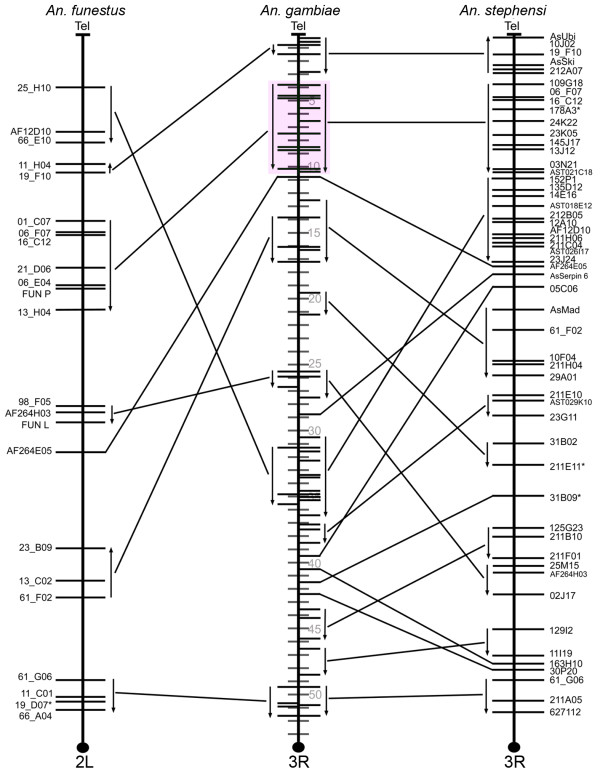
**Comparative mapping of chromosomal arms 3R of *An. gambiae*, 2L of *An. funestus*, and 3R of *An. stephensi***. Arrows denote oriented conserved gene orders. The largest block of genes with fully conserved order is highlighted with pink. Shaded divisions on the *An. gambiae *chromosomes denote the genomic coordinates in this species. The centromere regions are shown by black circles at the end of the arms.

**Figure 4:**
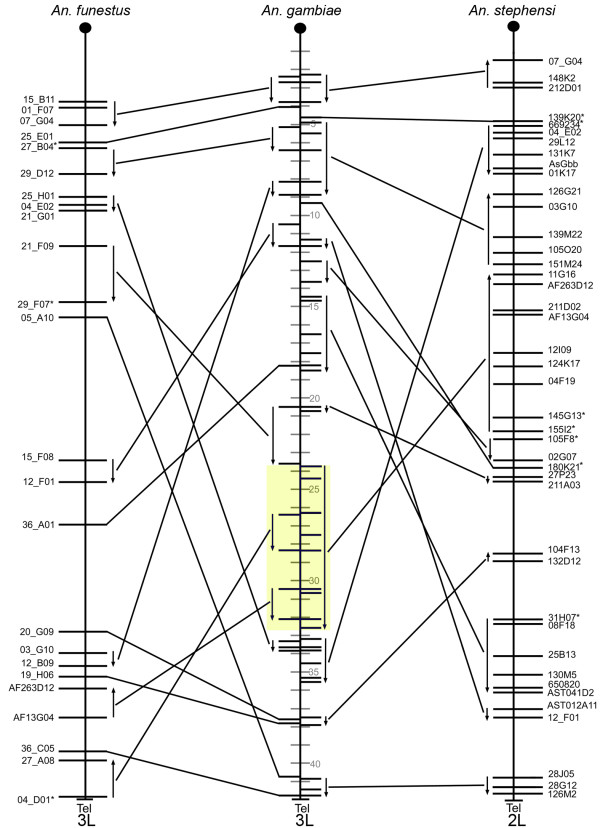
**Comparative mapping of chromosomal arms 3L of *An. gambiae*, 3L of *An. funestus*, and 2L of *An. stephensi***. Arrows denote oriented conserved gene orders. The largest block of genes with partly conserved order is highlighted with yellow. Shaded divisions on the *An. gambiae *chromosomes denote the genomic coordinates in this species. The centromere regions are shown by black circles at the end of the arms.

To avoid a lineage-specific bias in pair-wise analyses of gene orders, we estimated the chromosomal divergence among the mosquito species. Three-way comparative mapping can be very efficient in determining inversion distances among species. The cDNA and BAC clones physically and *in silico *mapped to polytene chromosomes of *An. funestus, An. stephensi*, and *An. gambiae *[11] were used to identify conserved gene orders among the three species (Additional file 1). The comparison of the physical maps of these species has identified the whole-arm translocations and paracentric inversions and detected no pericentric inversions or partial-arm translocations (Figures 1, 2, 3 and 4). Therefore we were able to determine the arm homology among species [11]. Accordingly, chromosome X (Additional file 2) and arm 2R (Figure 1) are homologous across all three species. The 2L arm of *An. gambiae *corresponds to the 3R of *An. funestus *and the 3L of *An. stephensi *(Figure 2). The 2L arm of *An. funestus *corresponds to the 3R arms of *An. gambiae *and *An. stephensi *(Figure 3). The 2L arm of *An. stephensi *corresponds to the 3L arms of *An. funestus *and *An. gambiae *(Figure 4). We have calculated inversion distances among *An. stephensi, An. gambiae*, and *An. funestus *based on locations of 87 common autosomal DNA markers in all three species (Additional file 3). This comparison has been done at the ~2.42-Mb level of resolution using the Multiple Genome Rearrangements (MRG) program (signed option) [25]. The MGR program has estimated 51 fixed inversions between *An. stephensi *and *An. gambiae*, 54 fixed inversions between *An. stephensi *and *An. funestus*, and 50 fixed inversions between *An. gambiae *and *An. funestus*. We also used the Genome Rearrangements In Man and Mouse (GRIMM) program without assuming directionality of the markers (unsigned option) to perform a pair-wise analysis of rearrangements [26]. The GRIMM program calculated 30 fixed inversions between *An. stephensi *and *An. gambiae*, 35 fixed inversions between *An. stephensi *and *An. funestus*, and 34 fixed inversions between *An. gambiae *and *An. funestus*. These data indicate that the three species have approximately equal chromosomal divergence from each other.

### Presence of similar sets of genes in polymorphic inversions of *An. gambiae, An. stephensi*, and *An. funestus*

**Figure 5:**
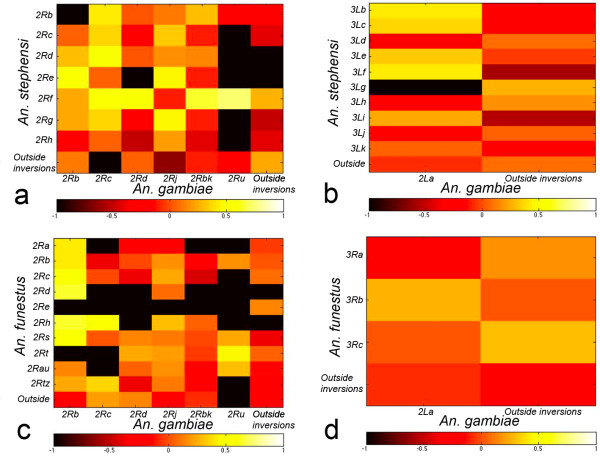
**Heat plots showing where there are more (and less) shared genes than would be expected, under pure chance between inversions**. Inversions on 2R (a) and 2L (b) arms of *An. gambiae *and *An. stephensi *and on the 2R (c) and 2L (d) arms of *An. gambiae *and *An. funestus *are shown. Light-yellow and black colors indicate nonrandom presence and absence of the same markers within inversions. Orange color indicates the random occurrence of markers.

**Figure 6:**
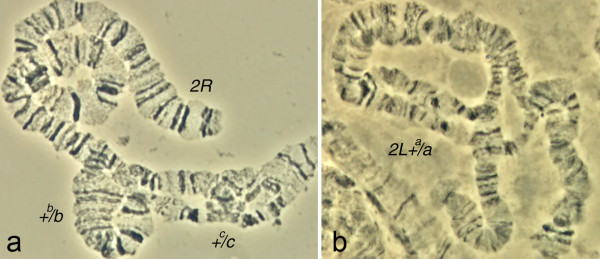
**Polymorphic inversions on chromosome 2 of *An. gambiae***. The heterozygote polymorphic inversions *2Rbc *(a) and *2La *(b) are shown.

The presence of common genes within inversions of homologous chromosomal arms could indicate that natural selection favors certain adaptive gene combinations when different species are exposed to similar environments. We tested for the presence or absence of physically and *in silico *mapped cDNA and BAC clones, which contained genes, in common polymorphic inversions of three mosquito species [14,16,18,27] at ~1-Mb level of resolution (Figures 1, 2, 3 and 4). In the previous study, we performed a test on the uniformity of the marker distribution across the chromosomes in *An. gambiae, An. stephensi*, and *An. funestus *using the *Χ*^2 ^statistic [11]. The distribution of the DNA markers was shown to be uniform for each arm and each species. The observed number of shared genes in polymorphic inversions of *An. stephensi *and *An. gambiae *(Additional file 4), as well as of *An. funestus *and *An. gambiae *(Additional file 5), were compared to those that would be expected under pure chance. Under the hypothesis that the genes are distributed due to pure chance with respect to polymorphic inversions and to each other, identical markers would be randomly distributed across a pair of chromosome arms from different species. Our results rejected this hypothesis as we found cases of nonrandom clustering of markers within polymorphic inversions in different species. Figure 5 shows the heat plots for the test statistic: (*O*_*i,j *_*- E*_*i,j*_)^2^/*E*_*i,j*_, which demonstrate the difference between the observed and expected number of shared markers in each inversion of *An. gambiae *and *An. stephensi*, as well as *An. gambiae *and *An. funestus*. Simulated p-values were computed from Monte Carlo simulated distributions, based on our test statistic, by considering the number of simulated replicates which were larger than the observed statistics (Table 1). Additional file 6 shows the probabilities that the intensity rate exceeds one for shared genes between *An. stephensi *and *An. gambiae*. Figure 5a, b shows the corresponding intensity heat map (based on the test statistic (*O*_*i,j *_*- E*_*i,j*_)^2^/*E*_*i,j*_, which aids in visually assessing the locations of shared hot and cold spots. We define *γ*_*i,j *_as the estimated factor of increased gene sharage, over what we would expect at random (See Methods for details). Inferred values where *γ*_*i,j *_= 1 suggest the shared polymorphism is in line with what we might expect to see at random. On the 2R arm, we observed that marker pairs *2Rf *(in *An. stephensi*) and *2Ru *(in *An. gambiae*) correspond to an activity hotspot (indicated by the light-yellow color in Figure 5a) with posterior probability Pr(*γ*_*i,j *_> 1|Data) = 1. The level of increase over expectation is dramatic (6.23 times). Similarly, additional file 6, accompanied by Figure 5 c, d, details results for shared polymorphic inversions between *An. funestus *and *An. gambiae*. Again we observed on the 2R arm that marker pairs between *2Rt *(in *An. funestus*) and *2Ru *(in *An. gambiae*) determine a hotspot of shared polymorphisms (Pr(*γ*_*i,j *_> 1|Data) = 1, 6.22 times). Another example of nonrandomly shared genes is the small *2Rb *inversion of *An. gambiae *(Figure 6a) and the overlapping inversions *2Rd *and *2Rh *of *An. funestus *(Figures 1 and 5c). The large *2La *inversion is the only common inversion in *An. gambiae *on this arm (Figure 6b). We found from little to no co-occurrence of the same markers in this inversion and polymorphic inversions on 3R of *An. funestus *and 3L of *An. stephensi *(less yellow and more orange boxes) (Figures 2 and 5b, d). The results provide evidence that several polymorphic inversions at least on the 2R arm of *An. gambiae *nonrandomly share gene combinations with inversions of *An. stephensi *and *An. funestus*.

**Table 1:** P-values for testing the null that the genes are shared between inversions due to chance

	Asymptotic p-value	Simulated p-value
*An. gambiae*/*An. stephensi *2R/2R	< 0.00001	< 0.00001
*An. gambiae*/*An. stephensi *2L/3L	0.1142	0.0798
*An. gambiae*/*An. funestus *2R/2R	0.2328	0.1884
*An. gambiae*/*An. funestus *2L/3R	0.5711	0.4447

It is important to note that chromosomal arms 2R and 2L of *An. gambiae *had the most rapid reshuffling of gene order in evolution among autosomes. The mean length of conserved gene blocks is 1.3 Mb on 2R and 1.7 Mb on 2L (Figures 1 and 2). These lengths are much smaller than the sizes of the common polymorphic inversions: 12.5 Mb (*2Rj*, 813 genes) [28], 8.24 Mb (*2Rb*, 548 genes) (Figure 6a), 4.67 Mb (*2Rc*, 329 genes) (Figure 6a), 4.02 Mb (*2Ru*, 264 genes) [29], and 21.6 Mb (*2La*, 1,281 genes) (Figure 6b) [30]. In some cases, the presence of similar sets of genes within inversions in different species could be explained by preservation of ancestral gene orders. For example, all three markers mapped to inversion *2Ru *of *An. gambiae *and inversion *2Rf *of *An. stephensi *formed a conserved block (Figure 1). However, many of the common markers did not belong to conserved gene blocks. Moreover, we observed the reshuffled positions of small conserved blocks within polymorphic inversions in different species (e.g., inversions *2Rb *of *An. gambiae, 2Re of An. stephensi*, and *2Rd*/*2Rh *of *An. funestus *in Figure 1). Therefore, colocalization of the common markers within polymorphic inversions is not only a result of the ancestral gene order but also a result of new genes combinations independently originated in different species.

To support the conclusion that gene sharing is more common in inversions than outside inversions, we have formed a metric for comparing the outside regions to the inverted regions. Table 2 shows the ratio of average levels of gene sharing (as compared to expected under the null model), between the outside regions relative to the inversions. We notice that for all comparisons the outside regions have less shared genes than inversions, as shown by the expected posterior assessments of the ratios being less than 1. For all except the *An. gambiae*/*An. funestus *2L/3R comparisons, 95% posterior credible intervals are bounded above by 1, which demonstrate these results are significant. For *An. gambiae*/*An. funestus *2L/3R, the 95% interval has strong overlap with 1, which demonstrates the level of connectivity (as compared to expected) is similar between inverted and outside regions.

**Table 2:** The ratio of average levels of gene sharing (as compared to expected under the null model), between the outside regions relative to the inversions

	Posterior expectation	95% Credible interval
*An. gambiae*/*An. stephensi *2R/2R	0.32	(0.20, 0.48)
*An. gambiae*/*An. stephensi *2L/3L	0.54	(0.40, 0.71)
*An. gambiae*/*An. funestus *2R/2R	0.44	(0.28, 0.55)
*An. gambiae*/*An. funestus *2L/3R	0.94	(0.52, 1.65)

### Conservation and disruption of gene order in mosquito evolution

To determine possible differences among chromosomal arms in the tolerance to generating evolutionary breakpoints, we performed comparative analysis of gene order preservation across species. Conserved gene blocks were defined as regions with the same order and distance between at least two markers in two or more different species. For example, there were small conserved blocks (~1 Mb) on arm 2R (Figure 1) as well as large conserved blocks (up to 6-8 Mb) on arms 3R and 3L of *An. gambiae *(Figures 3 and 4). In this study, we identified two types of conserved blocks of genes: (i) blocks that were conserved among all three species (fully conserved blocks) and (ii) blocks that were conserved between two species, but were disrupted in the third species (partially conserved blocks). The largest fully conserved and disrupted gene blocks are shown in Figure 3 and Figure 4, respectively. For each of the four autosomal arms, we counted blocks that were conserved between only *An. gambiae *and *An. funestus, An. gambiae *and *An. stephensi*, and those that were conserved among all three species simultaneously. Given the most recent common ancestor between each of our species of interest, disrupted blocks have accumulated through time, accounting the current level of block disruption in our sample (see Additional file 7 for a schematic visualization of this process). We focus our attention on two primary features of the blocks: the number and length of such blocks. The level of block disruption was inferred using a compound Poisson process, in which the number of conserved blocks follows a Poisson distribution and the length of the chromosomal arms scales the rate of the process (see Methods for details). While we were primarily interested in the level of block disruption, and how it differs between groups, we explicitly model levels for fully and partially conserved blocks (conserved+disrupted), since the counts are higher for these sets of blocks, and yield better estimation properties. The rate parameters {*λ*_*j*_*, γ*_*j*_} measure the abundance or block counts, and lengths, respectively on arm *j *∈ {2R, 2L, 3R, 3L}.

We denote the *fitted *compound Poisson process rate parameters as {*λ*_*j*_^*(c+d)*^*, γ*_*j*_^*(c+d)*^} and {*λ*_*j*_^*(c)*^*, γ*_*j*_^*(c)*^}, where the superscripts (c+d) and (c) denote whether the rates were fitted to conserved+disrupted or conserved blocks.

**Figure 7:**
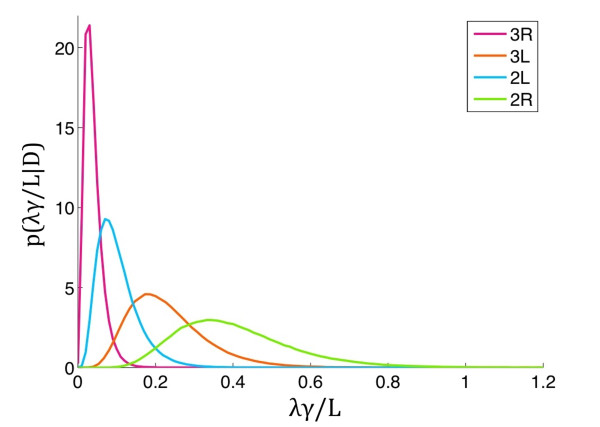
**Chromosome arm-specific differences in rates of accumulation of disrupted gene blocks**. X axis shows rates of accumulation of disrupted blocks per region length per unit length, z = (*λ*_*j*_^(*diff*) ^*γ*_*j*_^*(diff)*^)/L. Y axis shows a density of p(*z|D*); *p*--density, *D*--data.

The difference between these sets of rates (*λ*_*j*_^*(diff) *^*=λ*_*j*_^*(c+d) *^*- λ*_*j*_^*(c) *^and *γ*_*j*_^*(diff) *^*= γ*_*j*_^*(c+d) *^- *γ*_*j*_^*(c)*^) models the rates governing the disrupted blocks, which were then used to compare the levels of block disruption between groups. We report these rate parameters in conjunction with the combined summary (*λ*_*j*_^(*diff*) ^*γ*_*j*_^*(diff)*^)/*L*_*j*_, where *L*_*j *_is the total length of arm *j*. This combined summary has the interpretation of *blocks per region length per total length *(See Methods for further explanation). For both fitted processes, posterior summaries are displayed in Table 3. We demonstrated that the rate of accumulation of conserved and conserved+disrupted blocks was mildly higher for arms 2R and 3L. However, the lengths of the blocks were dramatically smaller on 2R than those found on other arms. For inferences of disrupted blocks, we considered the difference of these parameter pairings *λ*_*j*_^*(diff)*^ = *λ*_*j*_^*(c)*^*- λ*_*j*_^*(c+d) *^and (*γ*_*j*_^*-1*^)^(*diff*) ^= (*γ*_*j*_^(*c*)^)^-1 ^-(*γ*_*j*_^(*c+d*)^)^-1^. Strong overlap with zero, in each of the above parameter differences, indicated a negligible disruption rate. On the other hand, highly negative values indicated that the rates for the disrupted blocks were less than those for the conserved blocks. Conversely, large values suggested higher rates for the disrupted blocks. The analysis revealed that the 2R arm has the highest rate of accumulation of disrupted blocks per unit length, *λ*_*j*_^*(diff)*^, with a probability equal to 0.905 (Figure 7, Table 3). The effects for the other arms were less pronounced. Moreover, the 3R arm of *An. gambiae *and the homologous arms of the other species had the lowest *λ*_*j*_^*(diff) *^value with a probability of 0.5, and all identified gene blocks in this arm were preserved among *An. gambiae, An. funestus*, and *An. stephensi*. The data suggest that this chromosomal arm possesses evolutionary conserved breakpoint clusters and has low tolerance to generating new breakpoints.

**Table 3:** Expected parameter values and their associated 95% credible intervals shown for both conserved and disrupted blocks of each arm of An. gambiae*

Arm	***λ***_***j***_^**(*c+d*)**^	**(*γ***_***j***_^**(*c+d*)**^**)**^**-1**^	***λ***_***j***_^**(*c*)**^	**(*γ***_***j***_^**(*c*)**^**)**^**-1**^	***λ***_***j***_^**(*diff*)**^	**(*γ***_***j***_^***-1***^**)**^**(*diff*)**^
2R	0.248 (0.139, 0.387)	0.011 (0.006, 0.018)	0.149 (0.068, 0.260)	0.007 (0.004, 0.014)	-0.990 (-2.62, 0.058) 0.905	-0.004 (-0.012, 0.0001) 0.939

2L	0.166 (0.072, 0.298)	0.037 (0.018, 0.076)	0.103 (0.034, 0.212)	0.041 (0.016, 0.100)	-0.617 (-0.214, 0.084) 0.804	0.004 (-0.043, 0.06) 0.470

3R	0.115 (0.042, 0.224)	0.066 (0.029, 0.147)	0.116 (0.043, 0.225)	0.663 (0.029, 0.148)	-0.0004 (-0.013, 0.013) 0.500	-0.0002 (-0.091, 0.091) 0.500

3L	0.268 (0.134, 0.447)	0.030 (0.017, 0.055)	0.196 (0.085, 0.352)	0.294 (0.014, 0.060)	-0.72441 (-0.332, 0.138) 0.662	-0.000816 (-0.332, 0.138) 0.662

*The last numbers in the cells for *λ*_*j*_^(*diff*) ^and (*γ*_*j*_^*-1*^)^(*diff*) ^refer to probabilities.

## Discussion

### Parallel evolution of adaptive inversion polymorphisms in mosquito species

Natural selection seems to play a role in maintaining inversion polymorphisms on 2R and 2L of *An. gambiae *and their homologous arms in *An. stephensi *and *An. funestus *[14,16,17]. Our previous study has shown that chromosomal arms rich in polymorphic inversions (2R, 2L) have higher gene densities [11]. This observation confirmed the assumption that an inversion with fewer genes would have a smaller selective advantage [31]. Moreover, the study of gene ontology terms has provided evidence that 2L is enriched with genes involved in the structural integrity of a cuticle, while the 2R arm has an overrepresentation of genes involved in cellular response to stress (e.g., temperature, humidity) [11]. These data strongly support the role of natural selection in maintaining polymorphic inversions associated with adaptation of mosquitoes to the dry environment [14]. A study of larval ecology demonstrated that sympatric species *An. gambiae *and *An. funestus *inhabit a wide range of the same ecological settings in Cameroon [20]. We hypothesized that if polymorphic inversions in the distant species confer the same adaptations than similar sets of genes can be present within inversions of these species. The presence of similar sets of genes within independent inversions would imply the role of natural selection acting on similar genetic content of homologous chromosomal arms and creating parallel phenotypes of the evolutionary distant species. A theoretical model suggests that the probability of parallel evolution under natural selection is about two times bigger than that under neutrality [32]. Our results demonstrated that inversions on 2L of *An. gambiae*, 3L of *An. stephensi*, and 3R of *An. funestus *have almost random sets of genes (Figure 5b, d). However, we found that the several 2R inversions in *An. gambiae, An. stephensi*, and *An. funestus *do share common genes. The *2Rf *inversion of *An. stephensi *has an increased frequency in the urban environment [16] and nonrandomly share common genes with overlapping inversions *2Rc, 2Rd, 2Rbk*, and *2Ru *of *An. gambiae*. Another "urban" inversion in *An. stephensi, 2Rb*, had a gene homology to the inversion *2Rc *of *An. gambiae *(Figure 5a). In contrast, the *2Re *inversion of *An. stephensi *has an increased frequency in the rural environment [16] and nonrandomly shares common genes with inversion *2Rb *of *An. gambiae *and overlapping inversions *2Rd/2Rh *of *An. funestus*. This nonrandom distribution of markers is not only the result of preservation of ancestral gene order. In fact, we observed cases with extensively reshuffled gene orders within independently originated polymorphic inversions (Figure 1).

The gene shuffling is common in malaria mosquitoes [11], and these species are phylogenetically distant enough from each other [33,34] to have independently originated polymorphic inversions, which differ in chromosomal positions and size. The nonrandom presence of homologous genes within inversion *2Rb *of *An. gambiae *and inversion *2Rh *of *An. funestus *is especially interesting in the light of ecological adaptations associated with these inversions. The high frequency of the *2Rb *inversion of *An. gambiae *has been found strongly associated with increased degree of aridity. In contrast, the low frequencies of this inversion have been recorded in humid areas [35]. The *2Rh *inversion of *An. funestus *has a similar (although reverse) pattern of association with aridity. Correlation of the *2Rh *inversion with the higher vapor pressure has been demonstrated the strongest among all studied inversions of *An. funestus*. In contrast, the standard (*2Rh+*) arrangement has been found associated with the lower vapor pressure [21]. Thus, it is likely that natural selection favors adaptive gene combinations within polymorphic inversions on 2R when distantly related species are exposed to similar environmental pressures. The availability of these gene complexes would support long-term maintenance of polymorphic inversions. This knowledge could be useful for the discovery of genes responsible for an association of inversion polymorphisms with phenotypic variations in multiple species. If candidate genes were indentified within a polymorphic inversion in one species, the orthologous genes in another species likely play a similar role in adaptation if they are captured by a polymorphic inversion involved in the parallel adaptation. Future studies should identify specific alleles associated with parallel adaptation of species of subgenus *Cellia*.

### Arm-specific tolerance to disruption of gene order

We hypothesized that the arm-specific tolerance to chromosomal breakage could be responsible for the nonuniform distribution of inversions in autosomes. The comparative analysis of conserved and disrupted gene blocks in chromosomal arms across the three species provided evidence that 2R is more tolerant to disrupting gene orders and generating new evolutionary breakpoints than other arms (Figure 7). We observed that if a block on 2R was conserved between two mosquito species it was likely disrupted in the third species (Figure 1). In contrast, all identified gene blocks remain preserved on the 3R arm of *An. gambiae *and the homologous arms of the other species suggesting the existence of arm-specific constraints to breakage (Figure 4). These constraints could be controlled by negative selection acting against disruption of certain gene combinations. It is possible that slowly and rapidly evolving chromosomes may differ in sizes or abundance of coregulated gene clusters. Purifying selection against genomic rearrangements may preserve physical colocalization of coexpression clusters [23]. For example, clusters of genes deregulated in *trx *mutant *D. melanogaster *larvae are not uniformly distributed along the genome; 60% of them are located on chromosome 3L [36], which is a slowly-evolving arm in *Drosophila *[8,37]. Physically clustered genes may have shared regulatory regions, common expression pattern, and chromatin-level regulation, or they may represent clusters of essential genes [38]. Additionally, conservation of gene order in certain regions of the genome has been explained by long-range gene regulation [39]. If the 3R arm of *An. gambiae *is enriched in large functional gene clusters, then generating inversions on this arm or inserting a transgene into 3R will likely have a negative effect on mosquito fitness. Alternatively, the location of breakpoint clusters in only specific chromosomal regions of the 3R arm of *An. gambiae *and the homologous arms of the other species could be responsible for the preservation of the gene order in evolution. Indeed, a recent study has shown that fragility of certain regions rather than functional constraints plays the main role in nonuniform distribution of inversions in *Drosophila *chromosomes [24]. If this is the case in mosquitoes, than the distribution of breakpoints is lineage-specific on 2R and evolutionary conserved on the 3R arm of *An. gambiae *and the homologous arms of the other species. Our previous study has found that the 2R arm has the highest density of regions involved in segmental duplications that clustered in the breakpoint-rich zone of the arm. In contrast, the slower evolving 2L, 3R, and 3L, arms were enriched with matrix-attachment regions [11]. Future analyses of the genome sequence in different species will shed light on the exact mechanism of breakage in each individual chromosomal arm. Regardless of the mechanism, it is clear that new rearrangement breaks are more easily allowable on 2R than on other arms in different mosquito lineages, thus, contributing to the arm-specific differences in rates of chromosomal evolution in *Anopheles*.

## Conclusions

Our study demonstrated that polymorphic inversions on the 2R arm nonrandomly captured similar sets of genes in *An. gambiae, An. funestus*, and *An. stephensi*. This finding suggests that natural selection favors specific gene combinations within polymorphic inversions when distant species are exposed to similar environmental pressures. This knowledge could be useful for the discovery of genes responsible for an association of inversion polymorphisms with ecological adaptations in multiple species. We also found that the autosomal arms differ in their tolerance to disruption of syntenic blocks during mosquito evolution. The arm 2R has the highest level of inversion fixation among autosomal arms and the highest tolerance to disruption of syntenic blocks in evolution of subgenus *Cellia*. In contrast, the 3R arm of *An. gambiae *and homologous arms of the other species have few inversions and the lowest tolerance to disruption of syntenic blocks. All syntenic blocks found on this arm in two mosquito species were also preserved in the third species. Therefore, the distribution of breakpoint regions tends to be evolutionary conserved on slowly evolving arms and lineage-specific on rapidly evolving arms. These data support the chromosomal arm specificity in rates of gene order disruption during mosquito evolution.

## Methods

### Calculation of rearrangement distances

The sequences of cDNA and BAC clones physically mapped to polytene chromosomes of *An. funestus *and *An. stephensi *were used to identify homologous sequences in the *An. gambiae *genome through BLASTN and BLASTX algorithms available at VectorBase [40] in our previous study [11]. The MGR [25] and GRIMM [26] programs were utilized to calculate inversion distances among *An. gambiae, An. stephensi*, and *An. funestus *(Additional file 3). The MGR program is available at http://www.cs.ucsd.edu/groups/bioinformatics/MGR. The signed option of the MGR program was used. This program implements an algorithm that minimizes the sum of the rearrangements over all the edges of the phylogenetic tree [25]. The GRIMM program (unsigned option) was used to perform a pair-wise analysis of rearrangements [26]. GRIMM software uses the Hannenhalli and Pevzner algorithms for computing the minimum number of rearrangement events and for finding optimal scenarios for transforming one genome into another (http://grimm.ucsd.edu/GRIMM/). These algorithms use gene order information to estimate rearrangement distances.

### Chromosome preparation and visualization of inversions

Ovaries from the *An. gambiae *half-gravid females prefixed in Carnoy's fixative solution were dissected in 50% propionic acid under a Leica MZ6 dissection microscope (Leica Microsystems GmbH, Wetzlar, Germany). A cover slide was placed on the follicles and pressed to squash the cells. The banding pattern of polytene chromosomes was examined using Olympus CX-41 phase-contrast microscope (x1000) (Olympus America Inc., Melville, NY, USA). Slides with good chromosomal preparations were dipped in liquid nitrogen, then cover slips were removed and slides were dehydrated in 50%, 70%, 95%, and 100% ethanol. Preparations with polymorphic inversions were imaged with an Olympus Q-Color 5 camera and Q-Imaging software (Olympus America Inc., Melville, NY, USA). The chromosomal inversions were identified using a standard cytogenetic map for *An. gambiae *[41].

### Analysis of presence of common markers within polymorphic inversions of distant species

To assess the functional impact of shared genes on the genomic inversions, we begin by assessing the rate of accumulation of genes in inverted regions. If these genes have no bearing on inversions, we would expect the density of the genes located in these regions to be uniformly distributed throughout the genome. Our analysis centers on identifying regions of the genome that exhibit hot and cold spots for interaction. To test independence of markers, we compared the observed number of shared genes, in inverted regions, to those that would be expected under pure chance. Under the hypothesis that the genes are distributed due to pure chance, identically classified genes would be uniformly and independently distributed across a pair of chromosome arms (each from different species, which share the same genes).

Hence, if the inverted region, on each chromosome, accounts for the fraction  and  of the chromosome in species 1 and 2, respectively, for shared genes (*i,j*), then the probability that genes would be shared strictly by chance is. Therefore, given *N *total homologous gene markers, the expected number of shared genes, under pure chance, is.

For each inverted region, indexed by shared genes (*i,j*),(*O*_*i,j *_*- E*_*i,j*_)^2^/*E*_*i,j *_represents a discrepancy between the expected and observed number (O_*i,j*_) of shared genes. The statistic

has an *χ*^2 ^distribution when the sample size (*N*) is large. We use this framework to test if the genes are distributed in a manner consistent with the null hypothesis. While the test statistic has an asymptotic *χ*^2 ^distribution, the small sample sizes may yield inaccurate p-values. To simulate the distribution, we reposition each gene pair (*i,j*) on each of the corresponding gene arms, uniformly on each arm. For each stochastic realization, we count () the number of (stochastically repositioned) genes that fall in the regions corresponding to gene pair (*i,j*). Summing the test statistic () over each gene pair yields a random draw from the null distribution. We approximate the full distribution using 10,000 random draws and compare our test statistic, based on the observed quantities (O_*i,j*_, for all (*i,j*)-pairs). The fraction of random draws which exceed our observed test statistic closely approximates the true p-value, without any asymptotic assumptions.

To further study the observed arrangement of shared polymorphisms, we estimate the multiplicative rate at which gene sharing (for each (*i,j*)-pair) occurs above or below which is explainable at random. Below, we describe a Bayesian model for estimating these rates. Under the hypothesis that these regions arise uniformly and independently, the expected number of such shared genes is

where  and  are the respective fraction of the total genome length for species 1 and 2. Again, *N *represents the total number of homologous gene markers.

We use a Binomial sampling model, with mean , for measuring the abundance of shared genes (*i,j*), between species.

In the main text, we let (*γ*_*i,j *_= *λ*_*i,j *_+ *μ*_*i,j*_) for ease of explanation. We explain the parameters in this model below. We let *λ*_*i,j *_> 0 be a unit-less intensity parameter measuring the over/under abundance of shared genes, based solely on the interdependence of genes (*i,j*), and let *μ*_*i,j *_correspond to an additional random effect for the shared polymorphism being additionally hot (*μi,j *> 0), additionally cold (*μi,j *> 0), or neutral (*μ*_*i,j *_= 0). Explicitly, *λ*_*i,j *_models the average shared gene intensity, and *μ*_i,j _models any additional hot or cold intensity.

Since shared inversion regions are not necessarily independent (i.e. *λ*_*i,j*_and *λ*_*i,j*_, both depend on gene *j *in species 2), we factor the gene specific rate of influence by each region, and let

where  and  are marginal intensities corresponding to genes *i *and *j*, in their respective species. To create an identifiable model, we arbitrarily set . Hence, interpretation of the marginal intensity of gene connectivity is made solely through ratios of the form (*k*,*k*' ∈ {1,2}), which indicate the relative intensity of gene *i *(in species *k*), to gene *j *(in species *k*').

The random effects term *μ*_*i,j *_allows for additional variation in the intensity of shared polymorphisms, which can relate to extra cold (*μ*_*i,j *_< 0), or hot (*μ*_*i,j *_> 0) regions. This term accounts for the increase/decrease in shared polymorphisms, not accounted for by the averaging over gene intensities given by *λ*_*i,j*_.

The random effect model, for additional hot or cold intensities, follows as:

where *δ*(*condition*) is an indicator function, taking on the value 1 if the condition is true, and 0 otherwise. *N*(*μ*_*i,j*_|*μ*_H_,σ)δ({*μ*_*i,j*_,*μ*_*H*_} > 0) and *N*(*μ*_*i,j*_|*μ*_C_,σ)δ({*μ*_*i,j*_,*μ*_*C*_} < 0) represent Normal density functions, constrained to be positive or negative, depending on if the shared polymorphisms is exceedingly hot or cold. Under our scenario, mixing weights (*p*_0_, *p*_*H*_, p_c_) are unknown, as are the mean hot and cold temperatures: *μ*_*H *_and *μ*_*C*_, respectively.

Naturally, *p*_0 _+ *p*_H _+ *p*_C _= 1. While this model has a rich set of parameters, the overall model is identified due to the strong joint dependency in the parameter set. That is,  and  are identified by the average gene intensity, in species 1 and 2, respectively. The *μ*_*i,j*_'s are identified by the extreme over and under distribution of shared polymorphisms which are not explained by .

Given observed gene counts (*O*_*i,j*_) for each shared inversion region, the likelihood function follows as:

where , and Δ and *O *represent the matrices of intensity parameters, and associated counts of shared genes, for all (*i,j*) pairs. To estimate the unknown intensity parameters, we rely on a Bayesian inferential framework. Priors were selected as marginal reference priors. We describe these as follows.

For each *λ*_*i,j *_we let:.

We note that this prior is a global measure on the probability of observing shared polymorphisms between genes (*i,j*), given the fractional gene lengths . Such a structure will have nearly optimal frequency coverage properties (see [42] for details). For the mean hot and cold temperatures in the random effects compartment of the model, we specify *p*(*μ*_*H*_) = *p*(*μ*_*C*_)∝1. For the mixing weights, we let *p*(*p*_0_) = 0.99, and *p*(*p*_*H*_) = (*p*_*C*_) = 0.005. We have chosen this structure so that only very large deviations from the gene neutral model (*μ*_*i,j *_= 0) are declared hot or cold.

We focus on interpreting the joint quantities *γ*_*i,j *_= *λ*_*i,j *_+ *μ*_*i,j*_, through their proximity to 1. That is, values of *γ*_*i,j *_= *λ*_*i,j *_+ *μ*_*i,j *_close to 1 will support that genes are shared at random, for region (*i,j*). On the other hand, very high (and low) values will demonstrate and over (and under) accumulation of such genes. The relative over (or under) connectedness of individual genes can easily be made by considering the ratios 

Parameters were estimated using Markov Chain Monte Carlo (MCMC) [43]. Chains were burned in for 1,000,000 iterations, and used an additional 100,000 samples for estimation. Visual assessments of trace plots showed the chains had all reached stationarity. Multiple runs were made, starting from various distant locations in order to ensure convergence.

To analyze the level of gene sharing outside inverted regions, relative to that inside the inversion regions, we consider the average rate of connectivity (as compared to expected) of outside regions:

where *γ*_*i,out*_, *γ*_*out,j*_, and *γ*_*out,out *_denote the rates corresponding to outside the inversion regions on their respective arms. *N*^(1) ^and *N*^(2) ^denote the number of inverted regions on their respective arms. The sums are explicitly indexed over regions inside inversion regions which have connections between all outside regions. The average rate of connectivity (as compared to expected) within the inverted regions follows as:

By taking the ratio of these average rates:

we obtain a measure of how under (or over) connected the regions outside the inversions are compared to those within the inverted regions. Ratios near 1, demonstrate similar levels of connectivity, whereas under connectivity in the outside regions are demonstrated by ratios <1, and over connectivity in the outside regions are shown by ratios >1. Bayesian posterior assessments of these ratios are easily computed given our MCMC samples corresponding to each gene pair.

### Analysis of the rates of gene order disruption

We identified two types of conserved blocks of genes: (i) blocks that were conserved among all three species (fully conserved blocks) and (ii) blocks that were conserved between two species but were disrupted in the third species (partially conserved blocks). To analyze the rate of gene order disruption, between *An. stephensi *and *An. funestus*, with *An. gambiae*, it is necessary to consider the process that describes such genetic disruptions. Since these three separated species were descendants of some common species, at some point in time, each species had complete preservation of gene order with respect to the other two species. We denote this time by *t*_0 _= 0. This time represents the most recent time for which all three lineages can be mapped back to their most recent common ancestor. Through time, after these three species *speciated*, each of their respective genomes evolved at different rates, which resulted in sets of disruptions between the species groups. For discrete time points (*t*_0 _<*t*_1 _< ··· <*t*_*c*_) (*t*_*c *_is the current state of time), these disruptions appeared, resulting in a lower level of arm-specific preservation of gene order. This process of disruption accumulation is illustrated in Additional file 7, from which we observed that through time the length of the conserved blocks decrease with increased frequency. While we do not get to see this progression through time, we do observe the level of conservation at time *t*_*c*_. If this process were allowed to continue for an infinite amount of time, all blocks would eventually be disrupted. We note that our schematic only illustrates the basic idea, since inversions typically alter the location of the conserved blocks. In this analysis, we are concerned with how this process changes for each of the chromosome arms:{2R, 2L, 3R, 3L}. Since the disrupted blocks are accumulating through time, we account for both the number of conserved blocks and the length of each block at the current time (*t*_c_). We model the number and length of blocks using a compound Poisson process, where the number of conserved blocks follows a Poisson process and where the length of the chromosomal arm scales the rate of the process; a separate process governs each conserved block's length. Formally, for each arm *j *∈ {2R, 2L, 3R, 3L}, with total chromosome length *L*_*j*_, we model this process by

Where the number of observed blocks (*N*(*L*_*j*_)) follows a Poisson process, and *b*_*i,j *_denotes each individual block length and *R*_*j *_denotes the total length of conserved blocks on arm *j *(either fully conserved blocks, partially conserved blocks or both simultaneously), which is computed by the sum of individual block lengths on arm *j*. Specifically, we let

where the arm-specific parameters *λ*_*j *_and *γ*_*j *_define rates for the counting and length process, respectively. For the counting process, we have that

for the block length process, *γ*_*j *_is interpreted as the per-unit-length rate of accumulation of blocks. Hence, larger values of *γ*_*j *_indicate longer block lengths, whereas, for the block counting process, larger values of *λ*_*j *_will correspond to a higher quantity of blocks. It should be noted that the process is conditioned on the length of each chromosome (*L*_*j*_), which induces conditional independence. However, marginally *N*(*L*_*j*_), and b_*i,j *_are not assumed to be independent.

The individual lengths b_*i,j *_are scaled to the total arm length, so that

models the fraction of each arm that is occupied by a block. Alternatively, the length of all conserved blocks follows the distribution

so that *E*[*R*_*j*_] = *N*(*L*_*j*_)*L*_*j*_/*γ*_*j*_. We consider the simple summary of the compound process

that has the arm-specific interpretation of *blocks per region length per total length*.

On its own, the number of *blocks per region length *shows the density of conserved blocks within the conserved portion of each arm. Because each arm is of different length, we summarize our inferences by scaling this density to the *total length *of each arm. High values correspond to short blocks and/or high counts. Specifically, which rate is high (counts or lengths) is indistinguishable for our purposes. Hence, when summarizing results, it is convenient to think about *λ*_*j*_, *γ*_*j*_, and the above equation together. Our primary question is if the level of conserved disruptions accumulates at a rate different from that for fully conserved blocks. For this, we fit the process on both fully conserved and partially conserved blocks, and those pertaining to only fully conserved blocks. The parameter sets associated with each of the respective processes are denoted  and  representing the partially conserved and fully conserved processes. Other parameterizations are justifiable; however, due to the sparsity in disrupted blocks on some arms, we prefer this parameterization. For both processes, posterior summaries (rate expectations and 95% credible intervals) are displayed in Table 3.

## Abbreviations

GRIMM: Genome Rearrangements In Man and Mouse; MCMC: Markov Chain Monte Carlo; MGR: Multiple Genome Rearrangements.

## Authors' contributions

IVS designed research; IVS, AX, MVS, and SCL performed research; SCL, contributed new analytic tools; IVS, AX, MVS, and SCL analyzed data; IVS and SCL wrote the paper. All authors read and approved the final manuscript.

## Supplementary Material

Additional file 1 **Physically and *in silico *mapped DNA markers in the *An. gambiae, An. funestus*, and *An. stephensi *genomes**. The markers used for calculating inversion distances among *An. stephensi, An. gambiae*, and *An. funestus *are highlighted by yellow on 2R, green on 2L, teal on 3R and gray on 3L arms of *An. gambiae*. The genomic coordinates of polymorphic inversions are show for *2La *and *2Rj *in bold black, for *2Rb *in green bold font, for *2Rc *in red bold font, for *2Ru *in blue bold font. The genomic coordinates of overlapping polymorphic inversions are show for *2Rd *by underline and for *2Rbk *in italic font. Click here for file

Additional file 2 **Comparative mapping of the chromosomes X of *An. gambiae, An. funestus*, and *An. stephensi***. Arrows denote oriented conserved gene orders between *An. gambiae *and *An. stephensi*. Shaded divisions on the *An. gambiae *chromosome denote the genomic coordinates in this species. The centromere regions are shown by black circles at the end of the chromosomes. Click here for file

Additional file 3 **Calculation of rearrangement distances using the MGR and GRIMM programs**. Click here for file

Additional file 4 **Occurrence of DNA markers inside and outside of polymorphic inversions in *An. stephensi *and *An. gambiae***. Click here for file

Additional file 5 **Occurrence of DNA markers inside and outside of polymorphic inversions in *An. funestus *and *An. gambiae***. Click here for file

Additional file 6 **Presence of common markers within polymorphic inversions of distant species**. The probabilities (top numbers in cells) that the shared intensity is greater than 1, and shared intensity rate with corresponding 95% probability interval. Click here for file

Additional file 7 **Schematic illustration of the process of accumulation of disrupted gene orders**. S - *An. stephensi*, G - *An. gambiae*, F *- An. funestus*, t - time. Click here for file
