# Supplementary figures and images for: Arm-specific dynamics of chromosome evolution in malaria mosquitoes
Source: BMC Evol Biol. 2011 Apr 7;11:91. doi: 10.1186/1471-2148-11-91 (PMC3094232; doi:10.1186/1471-2148-11-91)

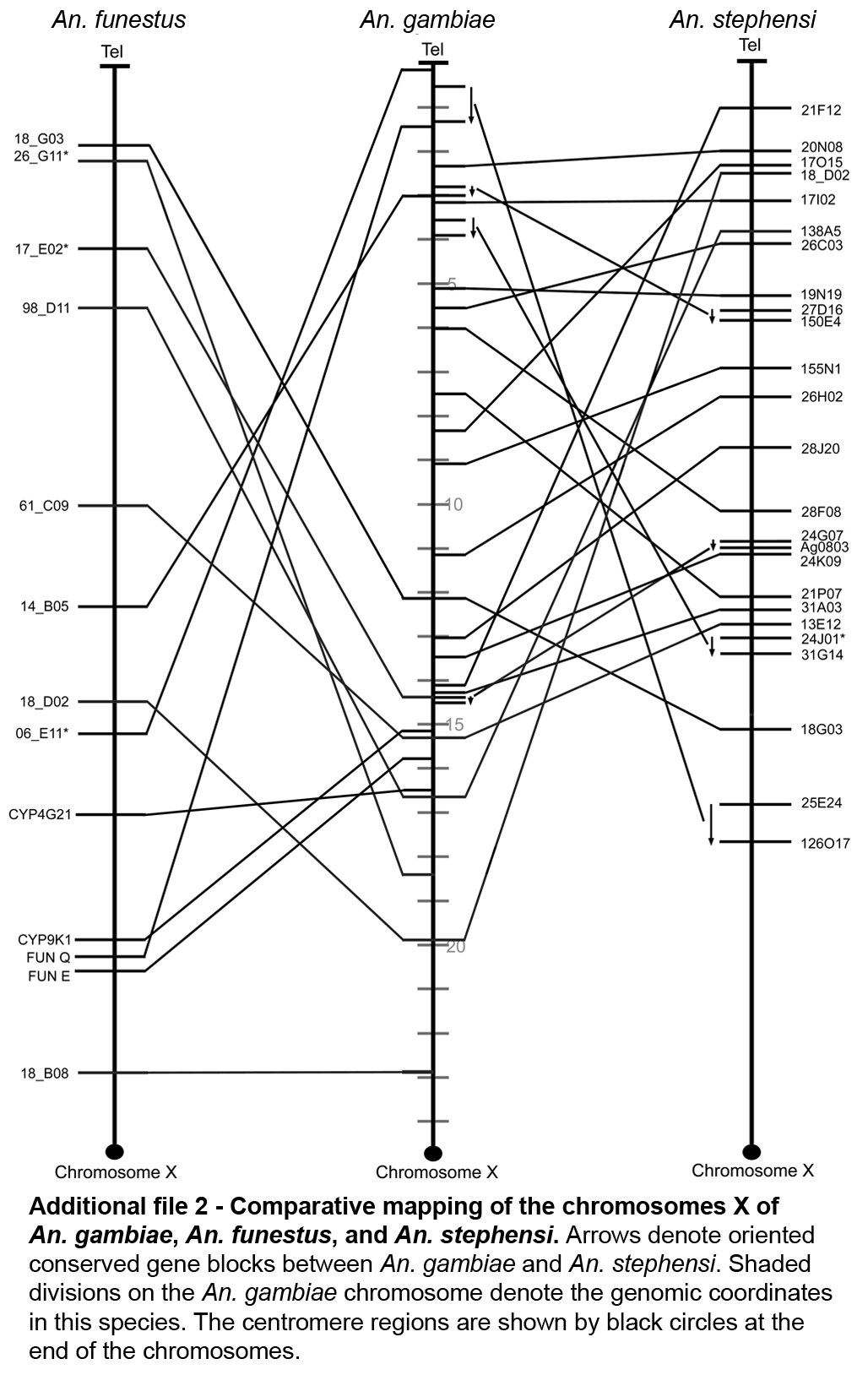

Supplement: Additional file 2 — Comparative mapping of the chromosomes X of An. gambiae, An. funestus, and An. stephensi. Arrows denote oriented conserved gene orders between An. gambiae and An. stephensi. Shaded divisions on the An. gambiae chromosome denote the genomic coordinates in this species. The centromere regions are shown by black circles at the end of the chromosomes. [file 1471-2148-11-91-S2.TIFF]

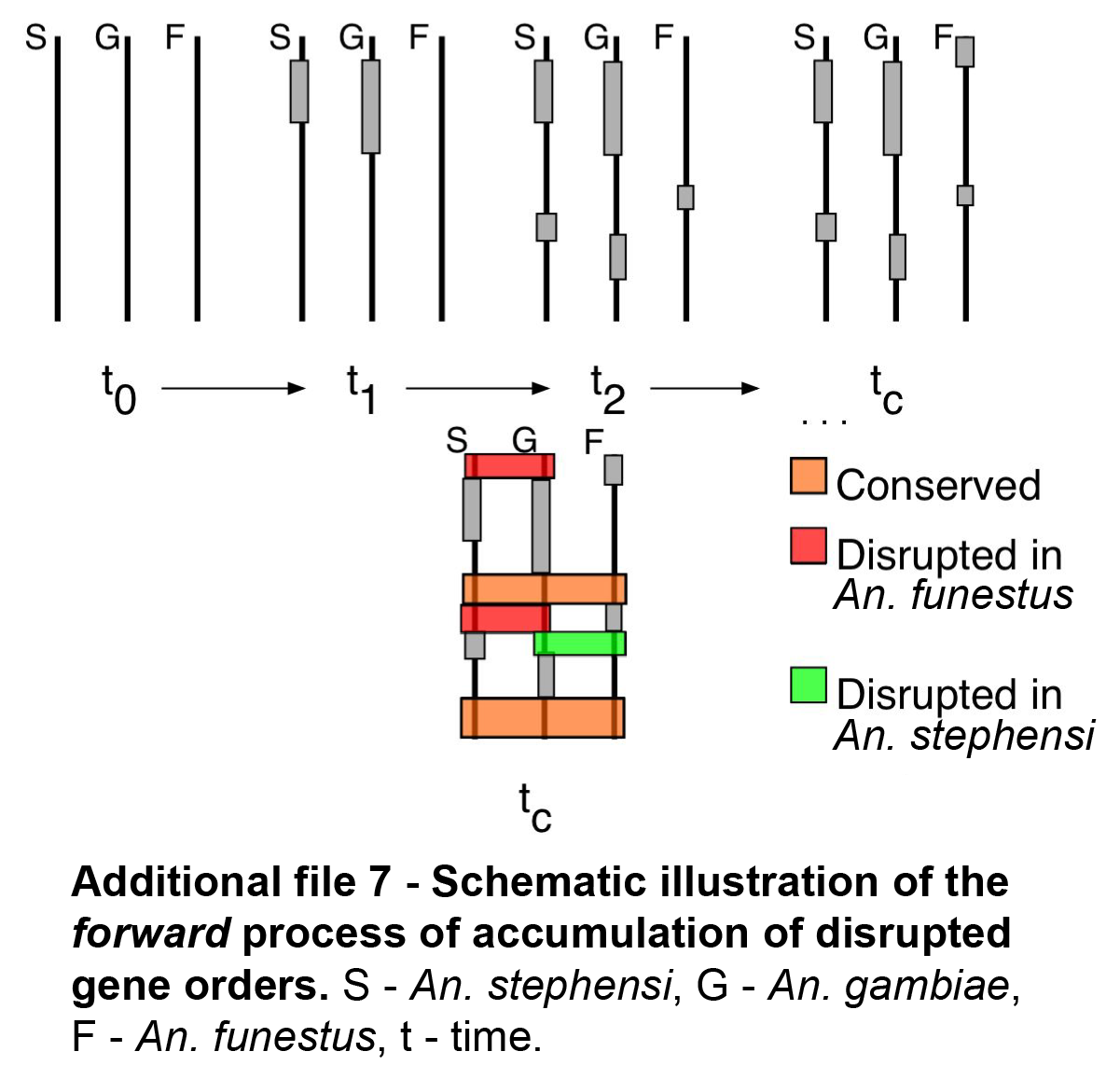

Supplement: Additional file 7 — Schematic illustration of the process of accumulation of disrupted gene orders. S - An. stephensi, G - An. gambiae, F - An. funestus, t - time. [file 1471-2148-11-91-S7.TIFF]
