# Supplementary material for: Arm-specific dynamics of chromosome evolution in malaria mosquitoes
Source: BMC Evol Biol. 2011 Apr 7;11:91. doi: 10.1186/1471-2148-11-91 (PMC3094232; doi:10.1186/1471-2148-11-91)
Supplement: Additional file 3 — Calculation of rearrangement distances using the MGR and GRIMM programs. [file 1471-2148-11-91-S3.DOCX]

**Additional file 3 - Calculation of rearrangement distances using the MGR and GRIMM programs.**

>*An. gambiae* 2R

1 2 3 4 5 6 7 8 9 10 11 12 13 14 15 16 17 18 19 20 21 22 23 24 25 26 27 28 29 30

>*An. stephensi* 2R

1 2 3 6 20 16 9 13 12 7 17 23 24 29 5 4 8 19 21 18 10 11 22 28 27 14 15 25 26 30

>*An. funestus* 2R

3 2 9 18 10 11 1 25 14 15 4 5 21 22 28 27 29 26 16 7 6 17 12 13 24 20 19 8 23 30

**ASCII representation of the unrooted tree recovered**

[+-----------------------13-------------------------](javascript:Edge(3,2))[An. funestus 2R](http://nbcr.sdsc.edu/GRIMM/mgr.cgi#Genome2)

[|](javascript:Edge(3,2))

[|](javascript:Edge(3,2)) [+-------------------11---------------------](javascript:Edge(3,1))[An. stephensi 2R](http://nbcr.sdsc.edu/GRIMM/mgr.cgi#Genome1)

[+-](javascript:Edge(3,2))[A3](http://nbcr.sdsc.edu/GRIMM/mgr.cgi#Genome3)

[+-------------------------14---------------------------](javascript:Edge(3,0))[An. gambiae 2R](http://nbcr.sdsc.edu/GRIMM/mgr.cgi#Genome0)

**Pairwise distance matrix of the input genomes (leaf nodes)**

Signed

|  | **An. gambiae 2R** | **An. stephensi 2R** | **An. funestus 2R** |
| --- | --- | --- | --- |
| **An. gambiae 2R** | [0](javascript:Edge(0,0)) | [21](javascript:Edge(0,1)) | [23](javascript:Edge(0,2)) |
| **An. stephensi 2R** | [21](javascript:Edge(1,0)) | [0](javascript:Edge(1,1)) | [23](javascript:Edge(1,2)) |
| **An. funestus 2R** | [23](javascript:Edge(2,0)) | [23](javascript:Edge(2,1)) | [0](javascript:Edge(2,2)) |

Unsigned

|  | **An. gambiae 2R** | **An. stephensi 2R** | **An. funestus 2R** |
| --- | --- | --- | --- |
| **An. gambiae 2R** | [0](javascript:Edge(0,0)) | [16](javascript:Edge(0,1)) | [15](javascript:Edge(0,2)) |
| **An. stephensi 2R** | [16](javascript:Edge(1,0)) | [0](javascript:Edge(1,1)) | [17](javascript:Edge(1,2)) |
| **An. funestus 2R** | [15](javascript:Edge(2,0)) | [17](javascript:Edge(2,1)) | [0](javascript:Edge(2,2)) |

>*An. gambiae* 2L

1 2 3 4 5 6 7 8 9 10 11 12 13 14 15 16 17 18 19 20 21

>*An. stephensi* 3L

1 2 3 10 6 4 11 12 13 5 7 8 14 15 9 16 17 18 19 20 21

>*An. funestus* 3R

12 13 10 15 14 8 1 2 3 5 6 7 11 4 9 16 17 18 19 20 21

**ASCII representation of the unrooted tree recovered**

[+----------------------------7-----------------------------](javascript:Edge(3,2))[An. funestus 3R](http://nbcr.sdsc.edu/GRIMM/mgr.cgi#Genome2)

[|](javascript:Edge(3,2))

[|](javascript:Edge(3,2)) [+-------------------5---------------------](javascript:Edge(3,1))[An. stephensi 3L](http://nbcr.sdsc.edu/GRIMM/mgr.cgi#Genome1)

[+-](javascript:Edge(3,2))[A3](http://nbcr.sdsc.edu/GRIMM/mgr.cgi#Genome3)

[+---------------4----------------](javascript:Edge(3,0))[An. gambiae 2L](http://nbcr.sdsc.edu/GRIMM/mgr.cgi#Genome0)

**Pairwise distance matrix of the input genomes (leaf nodes)**

Signed

|  | **An. gambiae 2L** | **An. stephensi 3L** | **An. funestus 3R** |
| --- | --- | --- | --- |
| **An. gambiae 2L** | [0](javascript:Edge(0,0)) | [9](javascript:Edge(0,1)) | [9](javascript:Edge(0,2)) |
| **An. stephensi 3L** | [9](javascript:Edge(1,0)) | [0](javascript:Edge(1,1)) | [11](javascript:Edge(1,2)) |
| **An. funestus 3R** | [9](javascript:Edge(2,0)) | [11](javascript:Edge(2,1)) | [0](javascript:Edge(2,2)) |

Unsigned

|  | **An. gambiae 2L** | **An. stephensi 3L** | **An. funestus 3R** |
| --- | --- | --- | --- |
| **An. gambiae 2L** | [0](javascript:Edge(0,0)) | [6](javascript:Edge(0,1)) | [7](javascript:Edge(0,2)) |
| **An. stephensi 3L** | [6](javascript:Edge(1,0)) | [0](javascript:Edge(1,1)) | [6](javascript:Edge(1,2)) |
| **An. funestus 3R** | [7](javascript:Edge(2,0)) | [6](javascript:Edge(2,1)) | [0](javascript:Edge(2,2)) |

>*An. gambiae* 3R

1 2 3 4 5 6 7 8 9 10 11 12 13 14 15 16 17 18

>*An. stephensi* 3R

2 1 3 4 5 6 7 8 9 16 17 10 11 12 13 14 15 18

>*An. funestus* 2L

16 17 2 1 3 4 5 6 7 8 9 14 15 10 13 12 11 18

**ASCII representation of the unrooted tree recovered**

[+----------------------------5-----------------------------](javascript:Edge(3,2))[An. funestus 2L](http://nbcr.sdsc.edu/GRIMM/mgr.cgi#Genome2)

[|](javascript:Edge(3,2))

[|](javascript:Edge(3,2)) [+----------2-----------](javascript:Edge(3,1))[An. stephensi 3R](http://nbcr.sdsc.edu/GRIMM/mgr.cgi#Genome1)

[+-](javascript:Edge(3,2))[A3](http://nbcr.sdsc.edu/GRIMM/mgr.cgi#Genome3)

[+----------------------4-----------------------](javascript:Edge(3,0))[An. gambiae 3R](http://nbcr.sdsc.edu/GRIMM/mgr.cgi#Genome0)

**Pairwise distance matrix of the input genomes (leaf nodes)**

Signed

|  | **An. gambiae 3R** | **An. stephensi 3R** | **An. funestus 2L** |
| --- | --- | --- | --- |
| **An. gambiae 3R** | [0](javascript:Edge(0,0)) | [6](javascript:Edge(0,1)) | [9](javascript:Edge(0,2)) |
| **An. stephensi 3R** | [6](javascript:Edge(1,0)) | [0](javascript:Edge(1,1)) | [7](javascript:Edge(1,2)) |
| **An. funestus 2L** | [9](javascript:Edge(2,0)) | [7](javascript:Edge(2,1)) | [0](javascript:Edge(2,2)) |

Unsigned

|  | **An. gambiae 3R** | **An. stephensi 3R** | **An. funestus 2L** |
| --- | --- | --- | --- |
| **An. gambiae 3R** | [0](javascript:Edge(0,0)) | [3](javascript:Edge(0,1)) | [5](javascript:Edge(0,2)) |
| **An. stephensi 3R** | [3](javascript:Edge(1,0)) | [0](javascript:Edge(1,1)) | [4](javascript:Edge(1,2)) |
| **An. funestus 2L** | [5](javascript:Edge(2,0)) | [4](javascript:Edge(2,1)) | [0](javascript:Edge(2,2)) |

>*An. gambiae* 3L

1 2 3 4 5 6 7 8 9 10 11 12 13 14 15 16 17 18

>*An. stephensi* 2L

2 1 14 15 16 6 5 4 3 13 12 11 10 9 17 8 7 18

>*An. funestus* 3L

1 2 3 4 14 15 16 9 7 8 17 5 6 13 12 18 11 10

**ASCII representation of the unrooted tree recovered**

[+-------------7--------------](javascript:Edge(3,2))[An. funestus 3L](http://nbcr.sdsc.edu/GRIMM/mgr.cgi#Genome2)

[|](javascript:Edge(3,2))

[|](javascript:Edge(3,2)) [+-------------------------13---------------------------](javascript:Edge(3,1))[An. stephensi 2L](http://nbcr.sdsc.edu/GRIMM/mgr.cgi#Genome1)

[+-](javascript:Edge(3,2))[A3](http://nbcr.sdsc.edu/GRIMM/mgr.cgi#Genome3)

[+--2----](javascript:Edge(3,0))[An. gambiae 3L](http://nbcr.sdsc.edu/GRIMM/mgr.cgi#Genome0)

**Pairwise distance matrix of the input genomes (leaf nodes)**

Signed

|  | **An. gambiae 3L** | **An. stephensi 2L** | **An. funestus 3L** |
| --- | --- | --- | --- |
| **An. gambiae 3L** | [0](javascript:Edge(0,0)) | [15](javascript:Edge(0,1)) | [9](javascript:Edge(0,2)) |
| **An. stephensi 2L** | [15](javascript:Edge(1,0)) | [0](javascript:Edge(1,1)) | [13](javascript:Edge(1,2)) |
| **An. funestus 3L** | [9](javascript:Edge(2,0)) | [13](javascript:Edge(2,1)) | [0](javascript:Edge(2,2)) |

Unsigned

|  | **An. gambiae 3L** | **An. stephensi 2L** | **An. funestus 3L** |
| --- | --- | --- | --- |
| **An. gambiae 3L** | [0](javascript:Edge(0,0)) | [5](javascript:Edge(0,1)) | [7](javascript:Edge(0,2)) |
| **An. stephensi 2L** | [5](javascript:Edge(1,0)) | [0](javascript:Edge(1,1)) | [8](javascript:Edge(1,2)) |
| **An. funestus 3L** | [7](javascript:Edge(2,0)) | [8](javascript:Edge(2,1)) | [0](javascript:Edge(2,2)) |
