# Supplementary material for: Arm-specific dynamics of chromosome evolution in malaria mosquitoes
Source: BMC Evol Biol. 2011 Apr 7;11:91. doi: 10.1186/1471-2148-11-91 (PMC3094232; doi:10.1186/1471-2148-11-91)
Supplement: Additional file 6 — Presence of common markers within polymorphic inversions of distant species. The probabilities (top numbers in cells) that the shared intensity is greater than 1, and shared intensity rate with corresponding 95% probability interval. [file 1471-2148-11-91-S6.DOCX]

**Additional file 6 - Presence of common markers within polymorphic inversions of distant species.***

| *An. stephensi* \ *An. gambiae* | | 2Rb | 2Rc | 2Rd | 2Rj | 2Rbk | 2Ru | Outside | Average |
| --- | --- | --- | --- | --- | --- | --- | --- | --- | --- |
| 2Rb | | 0.13  0.62  ( 0.56, 1.02) | 0.90  1.59  (0.76, 2.70) | 0.11  0.73  (0.36, 1.23) | 0.95  1.59  (0.90, 2.41) | 0.54  1.06  (0.57, 1.71) | 0.00  0.21  (0.03, 0.58) | 0.03  0.62  (0.33, 1.01) | 0.38 |
| 2Rc | | 0.43  0.99  (0.46, 1.78) | 0.77  1.37  (0.65, 2.45) | 0.04  0.61  (0.30, 1.07) | 0.83  1.35  (0.74, 2.19) | 0.31  0.90  (0.47, 1.49) | 0.00  0.18  (0.02, 0.51) | 0.01  0.53  (0.27, 0.89) | 0.34 |
| 2Rd | | 0.47  1.05  (0.42, 2.02) | 1.00  3.58  (1.59, 6.54) | 0.10  0.74  (0.28, 1.19) | 0.81  1.41  (0.67, 2.46) | 0.37  0.94  (0.42, 1.67) | 0.00  0.19  (0.02, 0.55) | 0.03  0.55  (0.24, 1.01) | 0.39 |
| 2Re | | 1.00  4.64  (3.60, 6.06) | 0.80  1.47  (0.64, 2.80) | 0.03  0.57  (0.29, 1.13) | 0.85  1.47  (0.71, 2.56) | 0.42  0.98  (0.45, 1.75) | 0.00  0.19  (0.02, 0.56) | 0.04  0.57  (0.26, 1.06) | 0.44 |
| 2Rf | | 1.00  2.93  (1.40, 5.16) | 1.00  4.01  (1.91, 6.96) | 1.00  2.12  (1.01, 4.19) | 0.83  1.91  ( 0.83, 3.33) | 1.00  3.28  (1.95, 5.82) | 1.00  6.23  (5.26, 7.66) | 0.91  1.57  (0.81, 2.65) | 0.96 |
| 2Rg | | 0.64  1.17  (0.60, 1.99) | 0.91  1.59  (0.82, 2.63) | 0.10  0.53  (0.38, 1.21) | 1.00  1.60  (1.06, 2.42) | 0.56  1.06  (0.61, 1.65) | 0.00  0.21  (0.03, 0.59) | 0.02  0.62  (0.35, 1.00) | 0.46 |
| 2Rh | | 0.08  0.67  (0.33, 1.16) | 0.34  0.92  (0.45, 1.56) | 0.00  0.42  (0.21, 0.71) | 0.33  0.92  (0.53, 1.41) | 0.02  0.61  (0.34, 0.96) | 0.00  0.12  (0.02, 0.34) | 0.00  0.36  (0.19, 0.59) | 0.11 |
| Outside | | 0.44  0.99  (0.49, 1.71) | 0.30  0.63  (0.37, 1.31) | 0.03  0.62  (0.31, 1.04) | 0.56  1.04  (0.54, 2.05) | 0.30  0.90  (0.51, 1.40) | 0.00  0.18  (0.02, 0.50) | 0.00  0.53  (0.29, 0.86) | 0.23 |
| Average | | 0.52 | 0.75 | 0.17 | 0.77 | 0.44 | 0.12 | 0.13 |  |
| *An. stephensi \ An. gambiae* | 2La+ | | | | | | Outside | Average |  |
| 3Lb | 0.9898  2.70  (1.19, 4.82) | | | | | | 0.3274  0.87  (0.25, 1.88) | 0.6586 |  |
| 3Lc | 0.9760  2.48  (1.00, 4.65) | | | | | | 0.3466  0.90  (0.26, 1.93) | 0.6613 |  |
| 3Ld | 0.4086  0.99  (0.13, 2.56) | | | | | | 0.7433  1.44  (0.49, 2.89) | 0.5759 |  |
| 3Le | 0.9766  2.18  (1.02, 3.83) | | | | | | 0.5189  1.06  (0.44, 1.95) | 0.7478 |  |
| 3Lf | 0.9877  2.88  (1.16, 5.27) | | | | | | 0.1194  0.56  (0.07, 1.50) | 0.5535 |  |
| 3Lg | 0.5901  1.69  (0.06, 5.90) | | | | | | 0.9054  2.64  (0.61, 6.17) | 0.7477 |  |
| 3Lh | 0.5336  1.23  (0.17, 3.21) | | | | | | 0.8557  1.78  (0.62, 3.67) | 0.6946 |  |
| 3Li | 0.9407  1.86  (0.84, 3.28) | | | | | | 0.0416  0.46  (0.10, 1.12) | 0.4911 |  |
| 3Lj | 0.4962  1.10  (0.25, 2.53) | | | | | | 0.7122  1.32  (0.51, 2.48) | 0.6042 |  |
| 3Lk | 0.7670  1.72  (0.38, 4.06) | | | | | | 0.4614  1.03  (0.23, 2.33) | 0.6142 |  |
| Outside | 0.4806  1.04  (0.39, 1.98) | | | | | | 0.7374  1.24  (0.64, 2.02) | 0.6090 |  |
| Average | 0.7406 | | | | | | 0.5245 |  |  |
| *An. funestus \ An. gambiae* | 2Rb | 2Rc | 2Rd | 2Rj | 2Rbk | 2Ru | Outside | Average |  |
| 2Ra | 1.00  2.96  (1.30, 4.01) | 0.00  0.08  (0.03, 0.18) | 0.00  0.12  (0.06, 0.21) | 0.00  0.40  (0.09, 0.69) | 0.00  0.09  (0.04, 0.16) | 0.05  0.39  (0.03, 1.67) | 0.40  0.70  (0.05, 1.37) | 0.20 |  |
| 2Rb | 1.00  2.45  (1.53, 3.59) | 0.12  0.66  (0.23, 1.34) | 0.43  0.98  (0.57, 1.51) | 0.94  1.41  (0.90, 2.05) | 0.08  0.72  (0.40, 1.14) | 0.36  0.91  (0.25, 1.42) | 0.35  0.81  (0.50, 1.19) | 0.46 |  |
| 2Rc | 1.00  3.36  (1.40, 5.04) | 0.09  0.73  (0.16, 5.86) | 0.18  0.77  (0.35, 1.36) | 0.60  1.11  (0.54, 1.85) | 0.03  0.27  (0.15, 1.01) | 0.07  0.57  (0.17, 1.23) | 0.05  0.64  (0.31, 1.07) | 0.28 |  |
| 2Rd | 1.00  4.87  (2.10, 7.07) | 0.0  0.10  (0.00, 0.41) | 0.00  0.10  (0.00, 0.28) | 0.96  1.53  (0.92, 3.77) | 0.00  0.06  (0.00, 0.20) | 0.30  1.78  (0.00, 6.45) | 0.27  0.32  (0.00, 1.22) | 0.36 |  |
| 2Re | 0.40  0.36  (0.00, 1.73) | 0.00  0.21  (0.00, .25) | 0.03  0.19  (0.00, .30) | 0.00  0.05  (0.00, 0.20) | 0.00  0.05  (0.00, 0.20) | 0.17  0.48  (0.00, 2.27) | 1.00  1.63  (1.03, 3.63) | 0.22 |  |
| 2Rh | 1.00  5.14  (2.31, 7.11) | 1.00  3.16  (2.33, 6.31) | 0.15  0.32  (0.28, 1.38) | 0.49  1.04  (0.44, 1.90) | 0.09  0.43  (0.11, 1.02) | 0.07  0.36  (0.14, 1.28) | 0.05  0.28  (0.14, 1.10) | 0.40 |  |
| 2Rs | 1.00  2.56  (1.48, 3.96) | 0.25  0.69  (0.23, 1.44) | 0.49  1.03  (0.56, 1.67) | 0.86  1.47  (0.89, 2.26) | 0.44  0.75  (0.39, 1.25) | 0.79  1.34  (0.45, 2.51) | 0.22  0.75  (0.46, 1.32) | 0.57 |  |
| 2Rt | 0.25  0.36  (0.25, 1.49) | 0.09  0.19  (0.18, 1.28) | 0.28  0.87  (0.42, 1.54) | 0.73  1.25  (0.65, 2.09) | 0.27  0.64  (0.30, 1.16) | 1  6.22  (5.37, 7.54) | 0.21  0.72  (0.37, 1.23) | 0.40 |  |
| 2Ru | 0.87  1.85  (0.80, 3.02) | 0.04  0.50  (0.16, 1.07) | 0.23  0.74  (0.38, 1.27) | 0.55  1.07  (0.59, 1.73) | 0.02  0.55  (0.27, 0.96) | 0.74  2.37  (0.42, 4.95) | 0.03  0.61  (0.33, 1.01) | 0.35 |  |
| 2Rz | 0.91  1.65  (0.80, 2.81) | 0.97  1.93  (0.98, 4.16) | 0.08  0.66  (0.30, 1.16) | 0.42  0.96  (0.45, 1.60) | 0.01  0.49  (0.22, 0.89) | 0.04  0.51  (0.14, 1.08) | 0.01  0.55  (0.26, 0.94) | 0.34 |  |
| Outside | 0.5618  1.29  (0.18, 3.35) | 0.8772  2.32  (0.53, 5.27) | 0.8370  1.70  (0.58, 3.53) | 0.4141  0.99  (0.21, 2.31) | 0.5965  1.18  (0.34, 2.53) | 0.3562  0.94  (0.03, 3.06) | 0.00  0.51  (0.26, 0.87) | 0.26 |  |
| Average | 0.8643 | 0.7140 | 0.6254 | 0.7886 | 0.5228 | 0.7173 | 0.13 |  |  |
| *An. funestus \ An. gambiae* | 2La | | | | | | Outside | Average |  |
| 3Ra | 0.2734  0.79  (0.18, 1.88) | | | | | | 0.8909  1.54  (0.76, 2.57) | 0.5821 |  |
| 3Rb | 0.9270  2.05  (0.78, 4.01) | | | | | | 0.6902  1.27  (0.49, 2.38) | 0.8086 |  |
| 3Rd | 0.7158  1.57  (0.37, 3.59) | | | | | | 0.9626  2.19  (0.93, 4.06) | 0.8392 |  |
| Outside | 0.5345  1.10  (0.37, 2.19) | | | | | | 0.2327  0.79  (0.31, 1.53) | 0.3836 |  |
| Average | 0.6127 | | | | | | 0.6941 |  |  |

* The probabilities (top numbers in cells) that the shared intensity is greater than 1 and shared intensity rate with corresponding 95% probability interval.
